# Zinc reduces epithelial barrier compromise induced by human seminal plasma

**DOI:** 10.1371/journal.pone.0170306

**Published:** 2017-03-09

**Authors:** James M. Mullin, Katherine M. Diguilio, Mary C. Valenzano, Rachael Deis, Sunil Thomas, E. Peter Zurbach, Shaheed Abdulhaqq, Luis J. Montaner

**Affiliations:** 1 Lankenau Institute for Medical Research, Wynnewood, PA, United States of America; 2 Department of Biology, Drexel University, Philadelphia, PA, United States of America; 3 Department of Chemistry, Saint Joseph’s University, Philadelphia, PA, United States of America; 4 The Wistar Institute, Philadelphia, PA, United States of America; Aix-Marseille University, FRANCE

## Abstract

Human semen has the potential to modulate the epithelial mucosal tissues it contacts, as seminal plasma (SP) is recognized to contain both pro- and anti-barrier components, yet its effects on epithelial barrier function are largely unknown. We addressed the role of human SP when exposed to the basal-lateral epithelial surface, a situation that would occur clinically with prior mechanical or disease-related injury of the human epithelial mucosal cell layers in contact with semen. The action of SP on claudins-2, -4, -5, and -7 expression, as well as on a target epithelium whose basolateral surface has been made accessible to SP, showed upregulation of claudins-4 and -5 in CACO-2 human epithelial cell layers, despite broad variance in SP-induced modulation of transepithelial electrical resistance and mannitol permeability. Upregulation of claudin-2 by SP also exhibited such variance by SP sample. We characterize individual effects on CACO-2 barrier function of nine factors known to be present abundantly in seminal plasma (zinc, EGF, citrate, spermine, fructose, urea, TGF, histone, inflammatory cytokines) to establish that zinc, spermine and fructose had significant potential to raise CACO-2 transepithelial resistance, whereas inflammatory cytokines and EGF decreased this measure of barrier function. The role of zinc as a dominant factor in determining higher levels of transepithelial resistance and lower levels of paracellular leak were confirmed by zinc chelation and exogenous zinc addition. As expected, SP presentation to the basolateral cell surface also caused a very dramatic yet transient elevation of pErk levels. Results suggest that increased zinc content in SP can compete against the barrier-compromising effect of negative modulators in SP when SP gains access to that epithelium’s basolateral surface. Prophylactic elevation of zinc in an epithelial cell layer prior to contact by SP may help to protect an epithelial barrier from invasion by SP-containing STD microbial pathogens such as HPV or HIV.

## Introduction

With the exception of pathogens introduced during intravenous drug use, the first obstacle that a pathogen faces when invading an organism is an epithelial cell layer. For microbial pathogens as diverse as viruses, bacteria or fungi, or even parasites such as dust mites, a considerable amount of evolutionary design has gone into transiting these barriers, and ingenious mechanisms have evolved (reviewed in[1–3]). The epithelial cell layer has developed a complex array of defenses ranging from passive approaches, like mucus secretion and acid microclimates, to more active measures, such as defensins, epithelial-derived cytokines and extravasation of white blood cells—a stratified system of defense that has been excellently reviewed recently for the female reproductive tract.[4, 5] Epithelial barrier defense in the specific context of HIV has also recently been reviewed.[6]

A pathogen can cross an epithelial barrier by invading an epithelial cell across its apical membrane and exiting the cell across its basolateral membrane, or by apically invading an epithelial cell, then killing the cell, thereby creating opportunity for systemic invasion by more pathogens, or by infecting cells “poking through” the barrier, such as dendritic cells, which can then “carry” the virus across the barrier.[7–10] However, certain pathogens have developed a different approach by directly binding to TJ components, such as specific claudin proteins, thereby enabling invasion of the epithelial cell, and then inducing junctional leakiness. This highly refined mechanism has been used by both bacterial and viral pathogens as seen, for example, in the etiology of *Clostridium perfringens* and hepatitis C.[11, 12] There are, however, other pathogens that enter the epithelial cell via interaction with non-junctional membrane proteins but still compromise the epithelial barrier by giving rise to junctional leakiness, a mechanism seemingly used by HIV and HPV.[13–15] A recent study by Abdulhaqq et al.[16] indicated that chronic unprotected sex is associated with changes in the female reproductive tract that may make HIV infection less likely. Frequent unprotected sex infers frequent exposure of the female reproductive mucosa to semen. Whether semen contains components that can induce changes to barrier function in the cervico-vaginal epithelium making HIV (and perhaps other viral STD) transmission less likely is unknown. We focus here specifically on SP contact with the basolateral surface of an epithelial layer, a situation that would occur whenever luminally presented SP comes into contact with an epithelium that has been damaged mechanically or by inflammation/infection.

## Materials and methods

### Collection and Handling of Seminal Plasma

De-identified, cryopreserved semen specimens were received under IRB exemption from the Andrology Laboratory of Penn Fertility Care, University of Pennsylvania. Up to 30 individual vials of semen were thawed at 37°C and then pooled into a single “sample” before aliquotting, refreezing and storage at -80°C. This was done to achieve a sufficient volume of SP to treat the cell layers in any given experiment. Samples were re-thawed at 37°C for use in this study, followed by centrifugation at 4°C to remove cells and thereby generate the SP used in this study. A given SP “sample,” therefore, is not from a single individual, but represents semen from as many as 30 individuals. Any such SP sample was used only once, and any excess was discarded. SP was never then refrozen. For use in experiments, SP was diluted 1:2 in culture medium and centrifuged at 3,000 rpm in an AccuSpin Micro R centrifuge at 4°C. Supernatant was then diluted a further 1:5 or 1:10 in culture medium before addition to cell layers.

**Fig 1:**
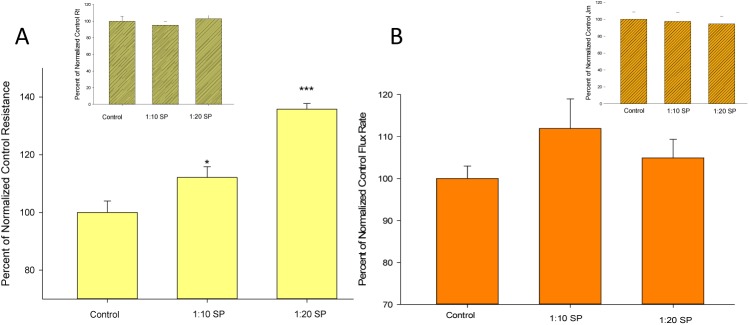
**Effect of seminal plasma on transepithelial electrical resistance (A) and transepithelial mannitol permeability (B) when presented to the basal-lateral cell surface.** Seminal plasma was diluted in culture medium at a 1:10 and 1:20 dilution level. Bars represent the mean ± standard error of n = 6 cell layers across 2 different experiments. *P = 0.05, *** P < 0.001 relative to control condition (Student’s t test, two-tailed). Inset bar graphs show the lack of a significant effect on R_t_ or J_m_ of 1:10 and 1:20 dilutions of SP when presented to the apical surface of CACO-2 cell layers.

**Fig 2:**
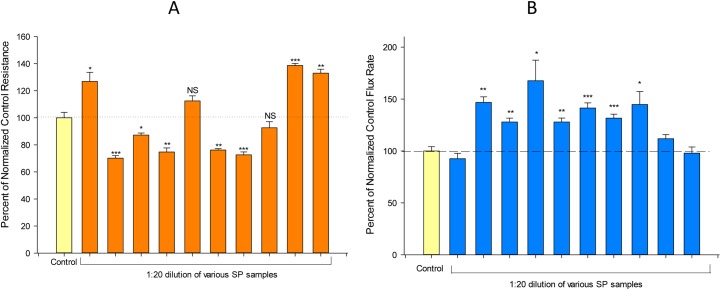
**Sample-to-sample variation in the effect of seminal plasma on transepithelial electrical resistance (A) and transepithelial mannitol permeability (B)**. Bars represent mean percentage change in R_t_ (panel A) or J_m_ (panel B) ± standard error for various samples of SP. Asterisks indicate level of statistical difference (Student’s t test, two-tailed), relative to control, for 4 cell layers (n = 12 for bar 2). * P < 0.05, ** P < 0.01, *** P < 0.001.

**Fig 3:**
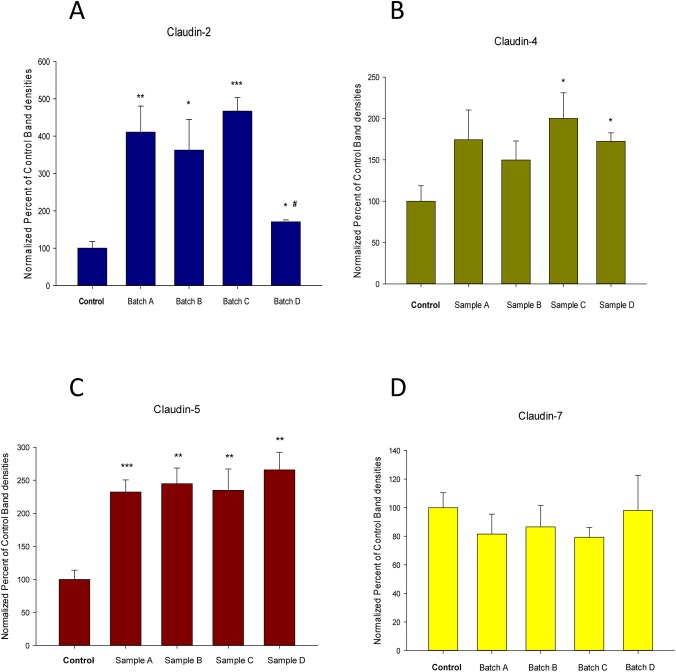
Sample-to-sample variation in the effect of seminal plasma on tight junctional proteins. Bars represent mean abundance ± standard error of specific tight junctional proteins (Claudin-2 [panel A], Claudin-4 [panel B], Claudin-5 [panel C] and Claudin-7 [panel D]) relative to the control condition, for 4 cell layers (3 cell layers for sample D). Asterisks indicate level of statistical difference (Student’s t test, two-tailed), relative to control: * P < 0.05, ** P < 0.01, *** P < 0.001. # P < 0.05 vs Samples A and C vs Sample D.

It merits emphasizing that each specific experiment (Figure) reported here used different (unique) batched SP samples. For example, the SP samples used in Fig 1 were distinct from those used in Fig 2, which were in turn distinct from those used in Fig 3, etc. This was done because of the small volume of individual semen samples, and the consequently small volume of even pooled (batched) SP samples. In addition, this study employed 4.2 cm^2^ cell layers, which allows for improved transepithelial measurements (less significant edge effects), but necessitates greater culture media volumes, and hence greater volume of SP samples. This, too, necessitated the use of different SP samples in different studies reported here.

### Cell culture

The CACO-2 BBE cell culture, an epithelial cell line derived from human colon adenocarcinoma,[17] was obtained from ATCC and used between passages 52 and 68. Upon confluence, cells were passaged on a weekly basis by trypsinization (0.25% trypsin and 2.2 mM EDTA [Corning Cellgro, Manassas, VA]) and were seeded at 7 x 10^5^ cells/Falcon 75-cm^2^ culture flask with 25 ml of Dulbecco-s Modified MEM (Minimum Essential Medium) (25 mM glucose[Corning Cellgro]) supplemented with 2mM L-glutamine (Corning Cellgro), 1% non-essential amino acids (Corning Cellgro), 1mM sodium pyruvate (Corning Cellgro) and 10% defined fetal bovine serum (HyClone, GE Healthcare Life Sciences, Pittsburgh, PA). Cultures were incubated at 37°C in 95% air-5% CO_2_ atmosphere.

### Treatment of epithelial cell layers with individual SP components

Nine-day post-confluent CACO-2 cell layers on Millipore PCF filters were exposed to varying concentrations of specific seminal plasma components in culture medium in the basal-lateral fluid compartment 24 hours prior to measurements of transepithelial electrical resistance (R_t_) and ^14^C-D-mannitol permeability (J_m_). Human recombinant proteins, TNF-α and IL1β, were obtained from PeproTech (Rocky Hill, NJ). IFN-γ was a product of Life Technologies (Frederick, MD). Zinc sulfate heptahydrate, histone from calf thymus, spermine, citric acid monohydrate, urea and fructose were products of Sigma-Aldrich (St. Louis, Mo.). Human recombinant EGF and TGF were products of Enzo Biochem (New York, NY) and PeproTech, respectively.

Whereas CACO-2 cultures 9 days post-seeding are not fully differentiated, barrier function is in fact near maximal.[18] Given our current study’s focus on exposure of the basal-lateral cell surface to SP, we are in fact modeling the effect of SP on an already compromised epithelium, one that allows SP penetration from the lumen into the interstitium, as would occur in an epithelium wounded mechanically or by proinflammatory cytokine damage. The epithelium in the immediate vicinity of that site of compromise would very likely not be in a completely differentiated state.

### Measurement of transepithelial electrical resistance and ^14^C-D-mannitol permeability

Cells were seeded into sterile Millipore Millicell polycarbonate (PCF) permeable supports (30 mm diameter with 0.4 μm pore size) on day 0 at a seeding density of 5 x 10^5^ cells/insert. Three or four sterile Millicell PCF inserts were placed into a 100 mm petri dish. On day 1, all cell layers were refed (2 ml apical/15 ml basolateral) with control medium containing 50 U/ml penicillin and 50 μg/ml streptomycin, followed by refeedings every 2–3 days until treatment. On the day of transepithelial experiments, the cell layers were refed with fresh control medium and allowed to incubate at 37°C for 1 to 1.5 hours prior to electrophysiological readings. Transepithelial potential difference, transepithelial electrical resistance (R_t_), and short-circuit current were measured using 1 sec, 40 μamp direct current pulses, and calculated using Ohm’s law as previously described.[19] As soon as electrical measurements were completed, the basolaterl medium was aspirated and replaced with 15 ml of medium containing 0.1 mM, 0.1 μCi/ml ^14^C-D-mannitol (PerkinElmer, Boston, MA) and incubated at 37°C. Triplicate basal-lateral medium samples (50 μl) were taken for liquid scintillation counting (LSC) for specific activity determination. Duplicate samples were taken from the apical medium at 60 and 120 min for LSC to determine flux rates. The media lost due to sampling from the apical compartment was replaced with fresh medium of the same sample volume. The flux rate (J_m_) (in cpm/min/cm^2^ and picomoles/min/cm^2^) was calculated for the ^14^C-D-mannitol diffusing across the cell layer.

### Immunoblot analyses of CACO-2 claudin proteins and pErk 1/2

Cells on Millipore PCF filters were washed 6X in 4°C phosphate-buffered saline (PBS) and then harvested by physical scraping into PBS, then centrifuged, and the cell pellet flash-frozen on dry ice. The pellet was thawed at 37°C in an SDS-containing lysis buffer, followed by sonication and ultra centrifugation. Supernatants were analyzed by PAGE using a 4–20% gradient Novex Tris-glycine gel (10% Tris-glycine gel for pErk) at 125V for 1 hr 45 min. Precision Plus Kaleidoscope Protein Standards (Bio-Rad Laboratories, Inc., Hercules, CA) were also included in each gel. Proteins were transferred at 30 V for 2 hr from the gel to a PVDF membrane. The membranes were then washed three times with PBS-T (0.3% Tween-20) for 10 min each and blocked with 5% milk/PBS-T (or 5% bovine serum albumin in PBST for pErk) for 1 hr at RT. Membranes were incubated with the specific primary antibody (Life Technologies for anti-claudins; Sigma Inc. for anti-pErk) at 1.0 μg /ml in 5% milk/PBS-T overnight at 4°C then 2 hr at RT (2 hrs at room temperature in the case of claudin-7). The membranes were washed with PBS-T 3X for 10 min each, then incubated with secondary antibody (rabbit anti-mouse or goat anti-rabbit IgG labeled with horseradish peroxidase [SouthernBiotech, Birmingham, AL] for 1 hr at RT). Membranes were washed with PBS-T (4X for 10 min each), then treated for 1 min with Western Lighting-ECL chemiluminescence reagents (PerkinElmer). The membranes were then exposed to HyBlot CL autoradiography film (Denville Scientific, Inc., South Plainfield, NJ), which was developed in a Kodak M35A X-OMAT processor. Band densities were quantified by densitometry (Bio-Rad Laboratories, Inc).

### Interleukin-8 assay

Interleukin-8 was measured in culture medium supplemented with seminal plasma using a BD Cytometric Bead Array (CBA) kit (BD Bioscience, San Jose, CA). The cytokine was analyzed following the manufacturer’s instructions using BD FACS Canto II Flow Cytometer and FCAP Array^**TM**^ Software (BD Bioscience). GraphPad Prism (GraphPad Software, Inc., La Jolla, CA) was used in the statistical analyses. Statistical significance was determined at 95% (*p*<0.05).

### Treatment of seminal plasma samples with Chelex-100 resin

SP was diluted 1:2 in culture medium, then centrifuged (10 mins, 200g) at room temperature to remove cells. The supernatant was collected, and Chelex-100 resin (Bio-Rad, 100–200 mesh, sodium form) was added at a ratio of 0.15 gms of resin per ml. The diluted SP was then rotated for 1 hr at 4°C, then centrifuged for 10 mins at 200g at room temperature to sediment the resin. The supernatant was then collected and diluted 1:10 in culture medium for a final SP dilution of 1:20. Note the mM concentrations of Ca and Mg in culture medium—and our method of chelating the 1:2 dilution followed by a 1:10 dilution in culture medium—means that chelation (of the 1:2 SP) has minimal effect on the final Ca and Mg levels in the 1:20 SP dilution. However, the very low Zn level of culture medium (only 10% FBS) means that chelation performed this way dramatically lowers Zn levels in the final 1:20 SP dilution.

### Analyses of zinc by atomic absorption spectroscopy

Atomic absorption measurements were performed using the PerkinElmer AAnalyst 800 atomic absorption spectrophotometer equipped with WinLab32 intuitive software. It utilizes a double beam optical system, solid-state detector, and deuterium background correction. A single slot air-acetylene 10 cm burner head was used for all measurements. Burner height and fuel gas flow were automatically optimized for each element using the WinLab32 software. Calibration standards in the range of 0.01 to 10.0 ppm were prepared by dilution of a 1000 ppm zinc AA standard (zinc metal in 3% nitric acid) obtained from Ricca Chemical Co. (Arlington, TX). Standards and samples were diluted with Millipore water (> 18.2 mega ohms), which also served as the blank. Aspirated standards and samples were measured in triplicate. For CACO-2 cells, zinc concentrations were normalized against total protein concentrations and expressed as μg/mg protein. For SP samples, zinc was measured after dilution in culture medium.

### Statistical treatment

All data are reported as the mean of n measurements ± standard error of the mean. Statistical significance was determined by employing the two-tailed, Student’s t test, comparisons being in all cases against a common control condition for that experiment. Statistical significance was applied to differences between groups where P < 0.05.

## Results

### Effects of basolateral presentation of seminal plasma on CACO-2 barrier function

The effect of SP on CACO-2 epithelial barrier function was evaluated by both R_t_, as well as by measurement of the transepithelial (paracellular) diffusion of ^14^C-D-mannitol (J_m_) across the same cell layers. Cell layers were exposed to 1:10 or 1:20 dilutions of SP on their basolateral surfaces as described in Materials and Methods. In two separate experiments, 24 hr exposure to SP was found to increase R_t_ significantly, although a statistically significant effect on J_m_ was not observed (Fig 1). The increase in R_t_ was significantly greater when a greater dilution of SP was used, the 1:10 dilution having a significantly lesser effect on Rt than a 1:20 dilution. This significantly greater effect of the lower SP concentration suggested that SP may contain barrier-supportive function as reflected by its effects on R_t_, as well as barrier-compromising components. The overall additive effect of such components on barrier function would depend on their relative concentrations in SP. The inset bar graphs in Fig 1 show the lack of a significant effect of SP on R_t_ or J_m_ when presented on the apical surface of the cell layer. The addition of SP to culture medium did not significantly alter culture medium osmolarity (control culture medium was 340 mOsm + 2 mOsm [SEM]; culture medium with a 1:10 dilution of SP was 342 mOsm + 3 mOsm [n = 3]).

### Major components of SP with individual actions on barrier function

Existing literature already identifies major components of SP with known modulatory activity on barrier function such as zinc, EGF, citrate, spermine, fructose, urea, and TGF (highlighted in Table 1) The dramatic elevation in concentration of some of these components—compared to their concentration in blood—is highlighted.

**Table 1:** Specific Components of Seminal Plasma in Substantial Excess Relative to Blood Plasma

Component	Seminal Plasma	Blood Plasma	Fold Increase From Blood
Citrate	18 mM^a^	0.13^b^	140X
Fructose	15 mM^a^	8^c^	1,800X
Urea	0.7 g/l^a^	0.2 g/l^d^	3.5X
Spermine	2.5 mM^a^	30 nM^e^	800,000X
Zinc	2 mM^a^	13.8 μM^f^	150X
EGF	48 ng/ml^a^	0.9 ng/ml^g^	50X
TGF	92 ng/ml^a^	5 ng/ml^h^	20X
IL-8	659 pg/ml^i^	29.3 pg/ml^j^	20X
Histone	16.9 μg/ml^a^*	0.06 ng/ml^k^	280,000X
TNF-α	140 pg/ml^l^	2.5 pg/ml^m^	55X
IL1-β	20 pg/ml^n^	15 pg/ml^m^	1.3X

Average levels from concentration ranges reported in the following biomedical literature

^a^ Kleine TJ, Gladfelter A, Lewis PN, Lewis SA. Histone-induced damage of a mammalian epithelium: the conductive effect. Am J Physiol 1995;May;268(5 Pt 1): C1114-25. Olsen J, Ramlau-Hansen CH. Dietary fats may impact semen quantity and quality. Asian J Androl 2012;Jul;14(4): 511–2. doi: 10.1038/aja.2012.52. Epub 2012 May 28. Jakobsen H, Rui H, Thomassen Y, Hald T, Purvis K. Polyamines and other accessory sex gland secretions in human seminal plasma 8 years after vasectomy. J

Reprod Fertil 1989;Sep;87(1): 39–45. Loras B, Vételé F, El Malki A, Rollet J, Soufir JC, Benahmed M. Seminal transforming growth factor-beta in normal and infertile men. Hum Reprod 1999;Jun;14(6): 1534–9. Hirata Y, Uchihashi M, Hazama M, Fujita T. Epidermal growth factor in human

seminal plasma. Horm Metab Res 1987;Jan;19(1): 35–7.

^b^ Fraenkl SA, Muser J, Groell R, Reinhard G, Orgul S, Flammer J, et al. Plasma Citrate Levels as a Potential Biomarker for Glaucoma. J Ocul Pharmacol Ther 2011;Dec;27(6): 577–80. doi: 10.1089/jop.2011.0062. Epub 2011 Sep 1.

^c^ Kawasaki T, Akanuma H, Yamanouchi T. Increased Fructose Concentrations in Blood and Urine in Patients With Diabetes. Diabetes Care 2002;25(2): 353–57.

^d^ Gomes M, Gonçalves A, Rocha E, Sá R, Alves A, Silva J, et al. Effect of in vitro exposure to lead chloride on semen quality and sperm DNA fragmentation. Zygote 2015;Jun;23(3): 384–93. doi: 10.1017/S0967199413000671. Epub 2014 Feb 13.

^e^ Igarashi K, Ueda S, Yoshida K, Kashiwagi K. Polyamines in Renal Failure. Amino Acids 2006;31(4): 477–83.

^f^ Gibson RS, Hess SY, Hotz C, Brown KH. Indicators of Zinc Status at the Population Level: A Review of the Evidence. BJN British Journal of Nutrition 2008;99: Suppl 3:S14-23. doi: 10.1017/S0007114508006818.

^g^ Hayashi T, Sakamoto S. Radioimmunoassay of human epidermal growth factor—hEGF levels in human body fluids. J Pharmacobiodyn 1988;Mar;11(3): 146–51.

^h^ Djurovic S, Schjetlein R, Wisløff F, Haugen G, Husby H, Berg K. Plasma Concentrations of Lp(a) Lipoprotein and TGF-Beta1 Are Altered in Preeclampsia. Clin Genet 1997;52(5): 371–76.

^I^ Orhan I, Onur R, Ilhan N, Ardiçoglu A. Seminal plasma cytokine levels in the diagnosis of chronic pelvic pain syndrome. Int J Urol 2001;Sep;8(9): 495–9.

^j^ Kleiner G, Marcuzzi A, Zanin V, Monasta L, Zauli G. Cytokine levels in the serum of healthy subjects. Mediators Inflamm 2013; 434010. doi: 10.1155/2013/434010.

^k^Ekaney ML, Otto GP, Sossdorf M, Sponholz C, Boehringer M, Loesche W, et al. Impact of plasma histones in human sepsis and their contribution to cellular injury and inflammation. Crit Care 2014;Sep 24;18(5): 543.

^l^Camus C, Matusali G, Bourry O, Mahe D, Aubry F, Bujan L, et al. Comparison of the effect of semen from HIV-infected and uninfected men on CD4+ T-cell infection. AIDS. 2016 May 15;30(8):1197–208. doi: 10.1097/QAD.0000000000001048.

^m^Lyke KE, Burges R, Cissoko Y, Sangare L, Dao M, Diarra I, Kone A, Harley R, et al. Serum levels of the proinflammatory cytokines interleukin-1 beta (IL-1beta), IL-6, IL-8, IL-10, tumor necrosis factor alpha, and IL-12(p70) in Malian children with severe Plasmodium falciparum malaria and matched uncomplicated malaria or healthy controls. Infect Immun. 2004 Oct;72(10):5630–7.

^n^ Huleihel M, Lunenfeld E, Levy A, Potashnik G, Glezerman M. Distinct expression levels of cytokines and soluble cytokine receptors in seminal plasma of fertile and infertile men. Fertil Steril. 1996 Jul;66(1):135–9.

* indicates a level of histone pertaining to semen, not seminal plasma.

To determine the potential contribution of these substances to the barrier effects produced by SP, we individually tested certain components across a concentration range likely present at the basal surface of an epithelium in vivo. For example, the 100 μM concentration of zinc used here is approximately the concentration of zinc that would be present in an SP sample diluted 1:20. As shown in Table 2, select concentrations of fructose, spermine and zinc exhibited barrier-enhancing activity as evidenced by increased R_t_ values. Histone reduced transepithelial mannitol leak (J_m_) significantly at one of the concentrations tested. Certain other SP components, such as a proinflammatory cytokine mixture (TNF-α, IFN-ϒ, IL1-β) and EGF, had barrier-compromising activity at both concentrations tested. Based on our prior published research concerning zinc-mediated epithelial barrier enhancement and tight junction modulation, [20] we focused on this particular substance in SP.

**Table 2:** Summary of effects of specific seminal plasma components on CACO-2 cell layer barrier function.

Component	% of Rt_c_	% of Jm_c_	n
***Zinc***			
0.1mM	130.7±7.6 (P = 0.004)	106.2±4.7 (NS)	8
0.02mM	90.1±3.8 (NS)	104.3±5.8 (NS)	8
***EGF***			
50ng/ml	55.0±3.0 (P<0.001)	121.4±12.6 (NS)	8
5ng/ml	60.9±1.3 (P<0.001)	108.1±5.8 (NS)	8
***Citrate***			
10mM	101.0±3.6 (NS)	90.8±5.6 (NS)	8
1mM	98.9±3.6 (NS)	107.8±10.4 (NS)	8-Rt/4-Jm
***Spermine***			
1.25mM	112.9±5.7 (NS)	110.8±13.0 (NS)	16
0.125mM	121.8±12.3 (P = 0.033)	90.5±6.5 (NS)	8
***Fructose***			
7.5mM	109.1±3.0 (P = 0.025)	100.5±4.4 (NS)	8
0.75mM	111.3±3.8 (P = 0.021)	104.5±5.4 (NS)	8
***Urea***			
0.35g/l	82.4±2.9 (P = 0.004)	107.4±8.4 (NS)	8
0.035g/l	96.8±2.4 (NS)	100.3±5.1 (NS)	8
***TGF***			
10ng/ml	93.6±1.9 (NS)	87.6±5.0 (NS)	8
1ng/ml	80.3±4.0 (P<0.001)	97.5±7.3 (NS)	8
***Histone***			
0.5μg/ml	101.1±3.3 (NS)	87.4±3.1 (P = 0.013)	12
0.05μg/ml	102.5±5.5 (NS)	93.2±6.0 (NS)	12
***Proinflammatory Cytokine Mixture (Cytomix)***^a^			
20/20/5 ng/ml	48.1±3.2 (P = 0.002)	125.9±4.4 (P = 0.005)	4
2/2/0.5 ng/ml	74.0±0.9 (P = 0.029)	104.4±3.1 (NS)	4

Nine-day post-confluent CACO-2 cell layers on Millipore PCF filters were exposed to 3 different concentrations of specific seminal plasma components in the basolateral fluid compartment 24 hrs prior to electrical measurements and radiotracer flux studies. Data shown represent the percentage of normalized control resistance and normalized control flux rate, and is expressed as the mean ± standard error for the n value listed in each case. P values are listed for instances of statistical significance (Student’s t-test [two-tailed] to at least the P<0.05 level). NS indicates non significance. Statistically significant barrier-enhancing effects are highlighted in blue, while significant barrier-compromising effects are highlighted in yellow.

^a^Cytomix refers to a mixture of Tumor Necrosis Factor-α, Interferon-ϒ and Interleukin-1-β at the concentrations indicated.

### Sample-to-sample variation in effects of SP on epithelial barrier function and claudin composition

In subsequent experiments using SP samples from different individuals but following the protocol of Fig 1, it was observed that different samples of SP had quite variable effects on barrier function—a variability that could be qualitative as well as quantitative. Specific samples of SP could have either an enhancing effect on R_t_, a compromising effect on R_t_, or no significant effect at all (Fig 2). Effects ranged from a 40% increase of R_t_ to decreases of R_t_ as great as 30%. The samples providing the greatest increase of R_t_ produced no effect on J_m_, whereas the samples that caused significant decrease of R_t_ also significantly increased J_m_. Therefore, the actual barrier effect of SP, in terms of its action on R_t_ and J_m_, depended upon the specific SP sample under study.

Corresponding to this difference in action upon R_t_ and J_m_, a sample-dependent effect of SP was likewise observed for effects on TJ protein composition. CACO-2 cell layers harvested after SP exposure and permeability studies, and analyzed as total cell lysates by western immunoblot, showed a pattern of changes in claudin proteins as a function of the specific SP sample. Across four distinct SP samples, no significant effect on claudin-7 levels were observed, but claudins-4 and -5 were significantly elevated by all SP samples (Fig 3). The SP effect on claudin-2 exhibited sample-to-sample variability, with three samples of SP inducing 400% increases in claudin-2, but a fourth sample exhibiting dramatically and significantly less of an effect on claudin-2.

### Seminal plasma sample variation regarding zinc and Interleukin-8

Focusing on two distinct components of SP that could play a role in barrier function, namely the trace metal zinc and the proinflammatory cytokine Interleukin-8 (IL-8), the sample-to-sample variability in the levels of these components of SP was determined.

**Fig 4:**
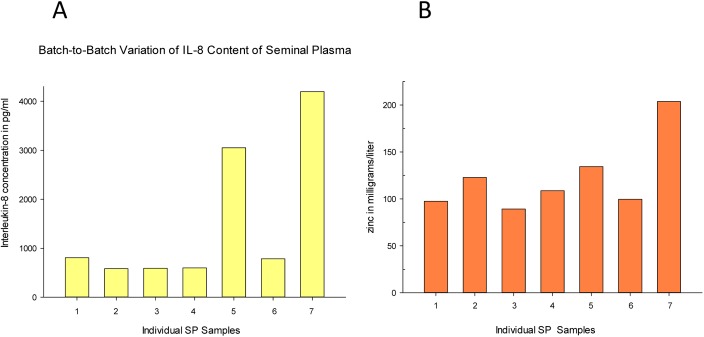
Sample-to-sample variation in Interleukin-8 (A) and zinc (B) concentrations in seminal plasma. Seminal plasma was diluted 1:20 in culture medium. IL-8 and zinc levels were measured as described in Materials and Methods. Each bar is the average of two (IL-8) or five (zinc) measurements with a variation of less than 5% of the mean.

As shown in Fig 4 (Panel A and B), variation in the content of IL-8 as high as four-fold was observed across seven samples of SP. Variation of total zinc content across the same seven samples was as great as 100%. It is worth noting that the sample containing the highest IL-8 activity also contained the highest zinc content.

In data not shown we tested for a correlation between zinc levels in specific samples of SP and the effect of such samples on epithelial barrier function. A statistically significant correlation was not found. Given the equally variable existence of many barrier-compromising substances in SP such as proinflammatory cytokines, it is not surprising that a single barrier-modulating component would neatly correlate—directly or inversely—with the overall action on barrier function.

### Reducing seminal plasma zinc content increases barrier function compromise

**Fig 5:**
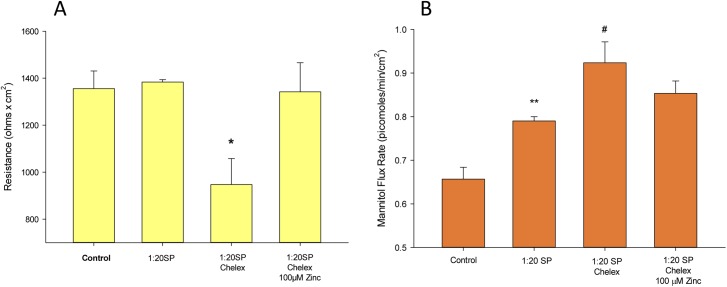
**Effect of zinc chelation on seminal plasma action on transepithelial electrical resistance (A) and transepithelial mannitol permeability (B).** Error bars represent standard error of the mean for n = 3 cell layers. * indicates P < 0.05, ** P < 0.01 for Student’s t-test, two-tailed, relative to control bar. # P = 0.05 relative to SP condition. Experiment was repeated 3 times with similar results.

Based on our own published findings and the growing published literature concerning zinc enhancement of epithelial barrier function generally,[19–22] as well as the enormous zinc content (and variability) of SP, we focused specifically on this particular SP component. When a specific sample of SP that produced no significant effect on R_t_ (at a 1:20 dilution) was subjected to zinc removal by chelation to Chelex-100 resin, it was observed that this extraction of zinc from the SP (prior to dilution in culture medium; see Materials and Methods) resulted in a modified SP that now exerted not only a significantly negative effect on R_t_ (35% decrease, P < 0.05), but also significantly increased the paracellular leak to ^14^C-D-mannitol (Fig 5). These effects of chelation on both R_t_ and J_m_ were both eliminated by re-addition of zinc (to the medium containing the chelated SP) to a 100 μM level (which earlier studies showed had a maximally enhancing effect on CACO-2 barrier function[19]). The specificity of this action for zinc was tested by addition of Ca (to an additional 2 mM); Mg (to an additional 1 mM); and Cu (to an additional 0.75 μM) back to the chelexed-SP-containing medium. However, none of these three divalent cations produced the reversal of effect on R_t_ or J_m_ that was observed by adding back zinc (data not shown), substantiating the positive role played specifically by zinc in the overall action of SP on barrier function in this epithelial model.

### Seminal plasma exposure induces Erk phosphorylation

**Fig 6:**
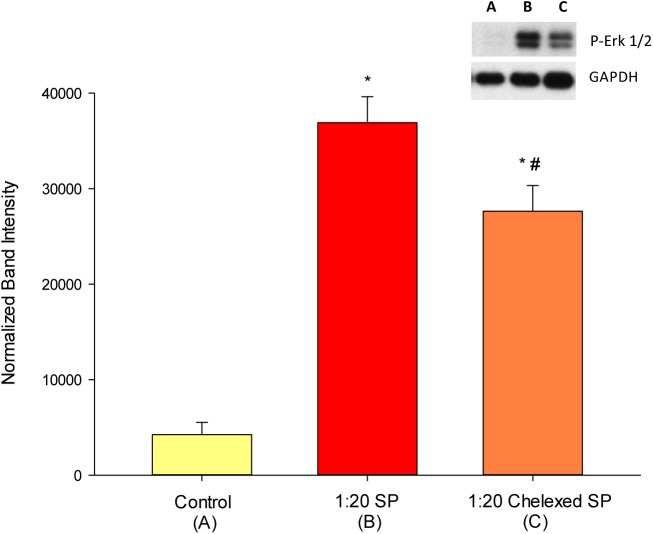
The increase in pErk levels after exposure of CACO-2 cell layers to SP. Seminal plasma was diluted in culture medium at a 1:20 dilution level before exposure to the basolateral surface of differentiated CACO-2 cell layers as described in Fig 1. Cell layers were exposed to either a 1:20 dilution of SP in culture medium, or a 1:20 dilution of SP treated with Chelex-100 for 15 minutes at 37°C. Cell layers were harvested as described in Materials and Methods, and whole cell lysates were analyzed by PAGE and western blot analyses for phosphorylated Erk 1/2, normalized to GAPDH. pErk band densities were quantitated by densitometry. Data points represent the mean of n = 3 cell layers ± standard error. * indicates P < 0.001 relative to the control condition; # indicates P < 0.05 relative to the SP condition (One Way ANOVA [Holm-Sidak method])

The MEK/Erk signaling pathway has been described as a common mediator of epithelial barrier and TJ leakiness.[2, 23, 24] It is thus noteworthy that exposure of CACO-2 cell layers to SP on their basolateral surface induced a strong 11-fold increase in pErk levels within 15 min of exposure to SP (Fig 6). Treating the SP with Chelex 100 resin, prior to exposure of the cell layers to the SP, reduced the magnitude of Erk 1/2 phosphorylation. The SP-induced phosphorylation of Erk was observed to have a rapid time course with pErk levels maximal at 15 mins after SP exposure, and declining to control levels by 4 hrs (data not shown).

## Discussion

This current study shows that in the CACO-2 epithelial model: 1) SP at fixed dilutions can have an enhancing effect on epithelial barrier properties when presented to the basolateral surface; 2) the effect is variable across individual SP samples, with certain samples producing a significant barrier-enhancing effect, while others produce a significant barrier-compromising effect, and certain others produce no significant effect at all, an effect that is reflected in actions upon tight junctional proteins; 3) removal of zinc from SP by chelation results in increased barrier-compromising activity by SP, irrespective of sample tested; 4) re-addition of zinc to the chelated SP restores barrier function; 5) this effect is specific for zinc, and does not hold for supplementation with Ca, Mg or Cu. These data suggest that zinc is one of the components, if not the major component, in SP that minimizes or prevents SP from significantly compromising epithelial barrier function if it contacts the basolateral epithelial surface. It remains to be determined whether zinc is directly counteracting a negative modulator of barrier function or is simply one of the major positive modulators of barrier function in SP. The fact that zinc within an approximately 10–100 μM concentration window can have a positive action on barrier function makes it possible that zinc can be elevated prophylactically at the site of a “target” epithelium that will come in contact with SP, in order to minimize barrier compromise coming from SP action on this epithelium. This could be critical when the zinc content of an ejaculate is low, which could then lead to paracellular leakiness in an epithelial tissue. It could be particularly an issue when zinc is low and certain cytokines in SP, such as IL-8, are elevated. IL-8 elevation in general is known to only be involved in HIV susceptibility but also in disease progression.[25, 26] Moreover, zinc deficiency is not uncommon in the HIV-infected population.[27]

A valid caveat on our data and the hypothesis they produce is that the further compromised barrier function produced by chelation treatment of SP (Fig 5)—and the amelioration of this compromise by the addition back of zinc—was evidenced much more in the R_t_ response than in the J_m_ response. Although addition of zinc back to SP did reduce the (paracellular) mannitol leak that elevated sharply with chelexed SP, this reduction was not significant to the P < 0.05 level as it was with the R_t_ recovery. One could argue, therefore, that the zinc-in-SP effect is much more significant for paracellular leak of simple salts, and question what is the extent of the biomedical significance of this phenomenon. However, we would point out that as yet we have not tested the SP and zinc-in-SP effects on large molecule transepithelial leak. This includes both charged and uncharged large molecules. This would entail running transepithelial flux studies with probes of 10kDa and 70kDa size, with and without charged groupings. The behavior of a very small uncharged probe such as D-mannitol, may not model leak pathways that could be opened or closed by SP or SP+zinc treatment respectively. This is a clear area for further investigation given its implications for epithelial tissue inflammation and infection.

This study has utilized CACO-2 gastrointestinal epithelial cell layers, a model epithelial barrier for which much information has been published.[28] It could be argued that the most medically applicable models with regard to STDs may in fact be vaginal or buccal/gingival epithelial cell layers to respectively model vaginal or oral sex activity. Future work by our group will test these findings in these models. Elevation of R_t_ by seminal plasma has in fact been reported for a uterine epithelial (HEC-1A) model.[29] However a colorectal model is not without merit here,[30, 31] and the findings obtained—the first of their kind—serve simply to demonstrate what could happen in terms of barrier compromise arising from SP contact of epithelial barriers in sexual activity. This being the first investigation of the effects of SP and zinc on epithelial barrier function, we chose the epithelial cell culture model—CACO-2—for which the greatest amount of published information existed. In addition, CACO-2’s high level of differentiation in cell culture afforded us a model with highly developed tight junctions and barrier function, and thus a very sensitive epithelial barrier model with which to probe SP effects. Although our findings with CACO-2 do not tell us what will happen regarding SP actions on unrelated epithelial tissues in vivo (e.g., oral, vaginal mucosae), these findings definitely alert us to what questions one may wish to address in future studies.

Apical presentation of SP to the epithelial model could be viewed as the most biomedically relevant application, since in sexual activity an ejaculate will first contact the luminal surface of an epithelial tissue. However, basolateral presentation of SP—as performed in this study—does mirror the *in vivo* situation of SP presentation to the apical surface of an epithelium whose barrier function has already been compromised mechanically or by the prior action of inflammation and/or certain pathogens.[31] Note that in this model we are not assuming that the barrier-compromising agents—or zinc itself—will efficiently diffuse through the TJs of a healthy epithelial barrier. Instead we are assuming that the epithelium has been compromised either mechanically or immunologically, and this site or sites of compromise provide the avenue for luminal SP to access the epithelial basal-lateral surface. The basal laterally mediated actions of luminally administered zinc were actually modeled in a gastrointestinal epithelium compromised pharmacologically by the action of cytochalasin-D.[32] Here the initially apically presented SP would simply diffuse through aberrantly leaky TJs or an actual multicellular “wound,” thereby accessing the basolateral surface at a reduced concentration (after mixing with interstitial fluid). This is the reason why the concentrations of components of SP tested in Table 2 are below the levels typically found in SP as modeled in this study.

This situation of an already compromised epithelial barrier and the contribution of zinc in SP may apply to a variety of clinical situations for various types of barriers. The gingival epithelium is frequently inflamed in periodontal disease, a condition even more acute in diabetics.[33, 34] Gingival inflammation—whether pathogen-induced or not—can result in barrier compromise[35] and be of significance in oral sex. Similar inflammation-based barrier compromise has been seen with uterine epithelial cell layers, a phenomenon that can be significant in vaginal sex.[36] A compromised epithelial barrier that is relevant to sexual activity can also occur by innate endocrinologic mechanisms totally unrelated to inflammation or pathophysiology.[37] As described in the context of HIV and HPV, prior infection of an epithelium can result in barrier compromise that in turn facilitates the penetration of additional pathogens.[38] All of the above scenarios have intrinsic significance for SP exposure and zinc levels as potential determinants for pathogen penetration of epithelial defenses, and therefore of STD transmissibility during and after sex. As described above, the ability of SP to further exacerbate barrier leak in low-zinc states could vary from one ejaculate sample to another. As a naturally occurring component in SP that opposes barrier compromise by other components of SP, it remains to be determined if zinc may have utility as a potential prophylactic therapeutic agent that can be used to minimize barrier compromise during sex. Of interest, males who have STD infections generally have low SP zinc levels.[39, 40]

The findings indicating that SP can enhance viral infection remains in controversy per the potential role of amyloid fibrils (e.g., SEVI) in transmission.[41] However, zinc and copper at micromolar concentrations have been shown to directly inhibit SEVI fibrillogenesis,[42] an action that could negatively affect HIV’s ability to infect in situ. The role of SEVI in viral infection may, however, be greater in *in vitro* models than *in vivo*.[43] When considering viruses other than HIV, zinc as a direct antiviral agent has shown efficacy against herpes simplex virus type 2 infection in mouse vaginal studies, but the high concentrations used (100–200 mM) resulted in sloughing of vaginal epithelia, thus undercutting its use as a contact-based, anti-viral agent.[44] Arens and Travis,[45] however, observed dramatically reduced herpes simplex virus type 2 activity at zinc concentrations as low as 15 mM.

In considering if the zinc in SP could be a determinant of whether SP has a protective or compromising effect regarding STD infectivity, our current study is not focused on the direct action of zinc on SP constituents (e.g., fibrillogenesis) nor on any possible negative action of zinc on a virus itself. Our current working hypothesis is that micromolar levels of zinc contacting the basolateral surface of the epithelial barrier induces a remodeling of the TJ complexes of that barrier that then result in reduced leak across the barrier. Whether zinc is having a direct beneficial effect on the TJ complexes, as we and others have seen in various epithelial models,[18–22, 46] and/or zinc is inhibiting/offsetting the damaging actions of other SP components on the epithelial barrier, will need added studies. The multicomponent nature of SP makes determining the molecular mechanisms for its action on TJ barriers quite complex.

We have, however, determined that SP induces a very rapid and dramatic upregulation of pErk 1/2, suggesting activation of the MEK/Erk signaling pathway may be involved in the observed effects on epithelial barrier function. While we did not also analyze for *total* Erk 1/2 in this study, the dramatic change in pErk in only a 15-minute incubation suggests this is in fact a phosphorylation-based change. The extensive literature describing MEK/Erk signaling inducing TJ leakiness[2, 23, 47, 48] suggests that the leak-inducing agent(s) in SP may here be mediating a leak through the MEK/Erk pathway. In fact, Erk-related change in TJ permeability can be positive as well as negative, depending upon the exact epithelial model in use.[49–51] Rapid phosphorylation of Erk after exposure to SP has been reported previously for endometrial and cervical carcinoma cells as well as vaginal epithelial cells.[52–54] Zinc has been reported to increase Erk phosphorylation in various cell types.[55, 56] This includes Erk phosphorylation in CACO-2 epithelia, the epithelial model in use in our study here.[57] The fact that Erk phosphorylation associates with leakiness, whereas zinc associates with junctional tightening AND Erk phosphorylation only indicates the complexity and poorly understood nature of the signaling mechanisms involved here. The MEK/Erk pathway at least appears to be one of the pathways by which SP transduces its effects on epithelial barriers and one pathway by which zinc can impart its barrier-protective action. Other well-described signaling pathways and elements regulating epithelial barrier function and TJs, such as Myosin Light Chain Kinase, protein kinase C isoforms, and calcium/calmodulin-dependent kinase, could figure just as prominently here in SP’s actions on barrier function and require future investigation. Involvement of other pathways is indicated by the fact that chelation treatment of the SP before exposure to the CACO-2 cell layers—a procedure that increases barrier leak brought about by SP (Fig 5)—reduced the level of Erk 1/2 phosphorylation caused by SP (Fig 6).

The discovery that zinc concentration in SP supports epithelial barrier function may provide a low-cost and safe adjunct approach to reducing STD transmissibility and infection. SP has been curiously described in the literature as being both facilitative to—and opposing—STD pathogen infection.[58, 59] If SP contains specific components that can compromise—and components that can enhance—the barrier function of an epithelial tissue, then the variable content of zinc may help explain the variance described in the literature to date. Identifying the components that enhance barrier function can form the basis for development of an eventual prophylactic use of these substances to decrease the likelihood of disease transmission during sexual activity.

## Abbreviations

EGF: epidermal growth factor
HPV: human papillomavirus
IFNϒ: interferon-ϒ
IL1β: interleukin-1β
I_sc_: short circuit current
J_m_: transepithelial mannitol diffusion rate
PBS: phosphate buffered saline
R_t_: transepithelial electrical resistance
SP: seminal plasma
STD: sexually transmitted disease
TGF: transforming growth factor
TJ: tight junction
TNF-α: tumor necrosis factor-α
